# Enhancing *z* Spin Generation in Trivial Spin Hall Materials for Scalable, Energy‐Efficient, Field‐Free, Complete Spin‐Orbit Torque Switching Applications

**DOI:** 10.1002/advs.202507581

**Published:** 2025-07-25

**Authors:** Qianbiao Liu, Lijun Zhu

**Affiliations:** ^1^ State Key Laboratory of Semiconductor Physics and Chip Technologies Institute of Semiconductors Chinese Academy of Sciences Beijing 100083 China; ^2^ Center of Materials Science and Optoelectronics Engineering University of Chinese Academy of Sciences Beijing 100049 China

**Keywords:** magnetization switching, spin current, spin Hall effect, spin‐orbit torque

## Abstract

Despite the remarkable efforts in the past two decades, it has remained a major challenge to achieve switching of perpendicularly magnetized spin‐orbit torque devices in a scalable, energy‐efficient, field‐free, integration‐friendly, and complete manner. Here, a giant enhancement of *z* spin generation in low‐resistivity spin Hall metal/FeCoB devices is reported by alloying the spin Hall metal Pt with Ti and by electric asymmetry engineering. The damping‐like spin torques of *z* spins and *y* spins are enhanced by 6 and 3 times relative to those of conventional Pt/FeCoB and enable complete, record‐low‐power, deterministic switching of FeCoB devices with strong perpendicular magnetic anisotropy and high coercivity. The Pt_75_Ti_25_/FeCoB heterostructure also exhibits relatively low resistivity, wafer‐scale uniform sputterdeposition on silicon oxide, good compatibility with magnetic tunnel junctions, and excellent thermal stability of exceeding 400 °C. These results unambiguously establish the Pt_75_Ti_25_/FeCoB as the most compelling candidate for solving the bottleneck of scalable, energy‐efficient, field‐free, integration‐friendly, and complete spin‐orbit torque switching technologies. This work also provides a universal strategy for developing high‐performance generators of *z*‐spin current and will stimulate the exploration of exotic spin currents by alloying “trivial” spin Hall materials.

## Graphical abstract

Z‐spin current has the potential to enable efficient electrical switching of perpendicular magnetization. This article reports a giant enhancement of *z* spin generation in low‐resistivity spin Hall metal/FeCoB devices by alloying Pt with Ti and by electric asymmetry engineering, providing a universal strategy for developing high‐performance generators of *z* spin current.

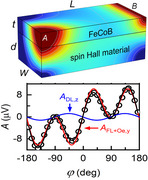

## 1 Introduction

Perpendicular magnetization is attractive for spin‐orbit torque (SOT)‐driven memory and computing with long data retention.^[^ ^1^ ^]^ However, after the two‐decade intensive efforts,^[^ ^2^, ^3^, ^4^, ^5^, ^6^, ^7^, ^8^, ^9^, ^10^, ^11^, ^12^, ^13^, ^14^, ^15^, ^16^, ^17^, ^18^, ^19^, ^20^, ^21^, ^22^, ^23^, ^24^, ^25^, ^26^, ^27^, ^28^, ^29^, ^30^, ^31^, ^32^, ^33^, ^34^, ^35^, ^36^, ^37^, ^38^, ^39^, ^40^, ^41^, ^42^, ^43^ ^]^ it has remained a challenge to switch a stable, uniform perpendicular magnetization by an in‐plane current in a scalable, energy‐efficient, magnetic‐field‐free, and integration‐friendly manner. For example, a large charge current and a large in‐plane magnetic field along the current direction (*x* direction) are typically required to switch a uniform perpendicular magnetization by the transverse spins (*y* spins, polarized in the *y* direction but flowing in the *z* direction).^[^ ^1^, ^2^ ^]^ While, as first proposed by Liu et al.^[^ ^39^ ^]^ in 2012 and later experimentally verified by several groups,^[^ ^6^, ^7^, ^24^, ^25^, ^36^, ^38^, ^40^, ^41^, ^42^, ^43^ ^]^ the in‐plane longitudinal assisting magnetic field can be applied easily by the dipole or exchange fields of a nearby in‐plane magnetic layer, the fast deterministic write via this scheme typically requires a current that is considerably higher than that can be afforded by the mainstream FinFET transistors (<100 µA) due to the current density of typically 10–100 MA cm^−2^ and the large channel thickness. The spin current of perpendicular spins (*z* spins, both polarized and flowing in the *z* direction)^[^ ^22^, ^23^, ^24^, ^25^, ^26^, ^27^, ^28^, ^29^, ^30^, ^31^, ^32^, ^33^, ^34^ ^]^ has the potential to enable, by itself or in combination with *y* spin current, field‐free switching of perpendicular magnetization. However, most discussions of the *z* spins have been limited to spin‐rotatory interfaces of magnetic multilayers^[^ ^23^, ^24^, ^25^ ^]^ that have low energy efficiency (see below) and crystallographically or magnetically low‐symmetry single crystals^[^ ^22^, ^26^, ^27^, ^28^, ^29^, ^30^, ^31^, ^32^, ^33^ ^]^ that can hardly be integrated into large‐scale complementary metal‐oxide‐semiconductor (CMOS) circuits. Spin current generation via the anomalous Hall effect and anisotropic magnetoresistance of perpendicular or titled magnetization has also been proposed^[^ ^44^, ^45^ ^]^ but has not been verified for magnetization switching. Importantly, most of the so‐called “field‐free” switching strategies can only yield incomplete switching of the magnetization and are incompatible with perpendicular FeCoB‐MgO magnetic tunnel junctions, which questions the practical usefulness of the strategies.

**Figure 1:**
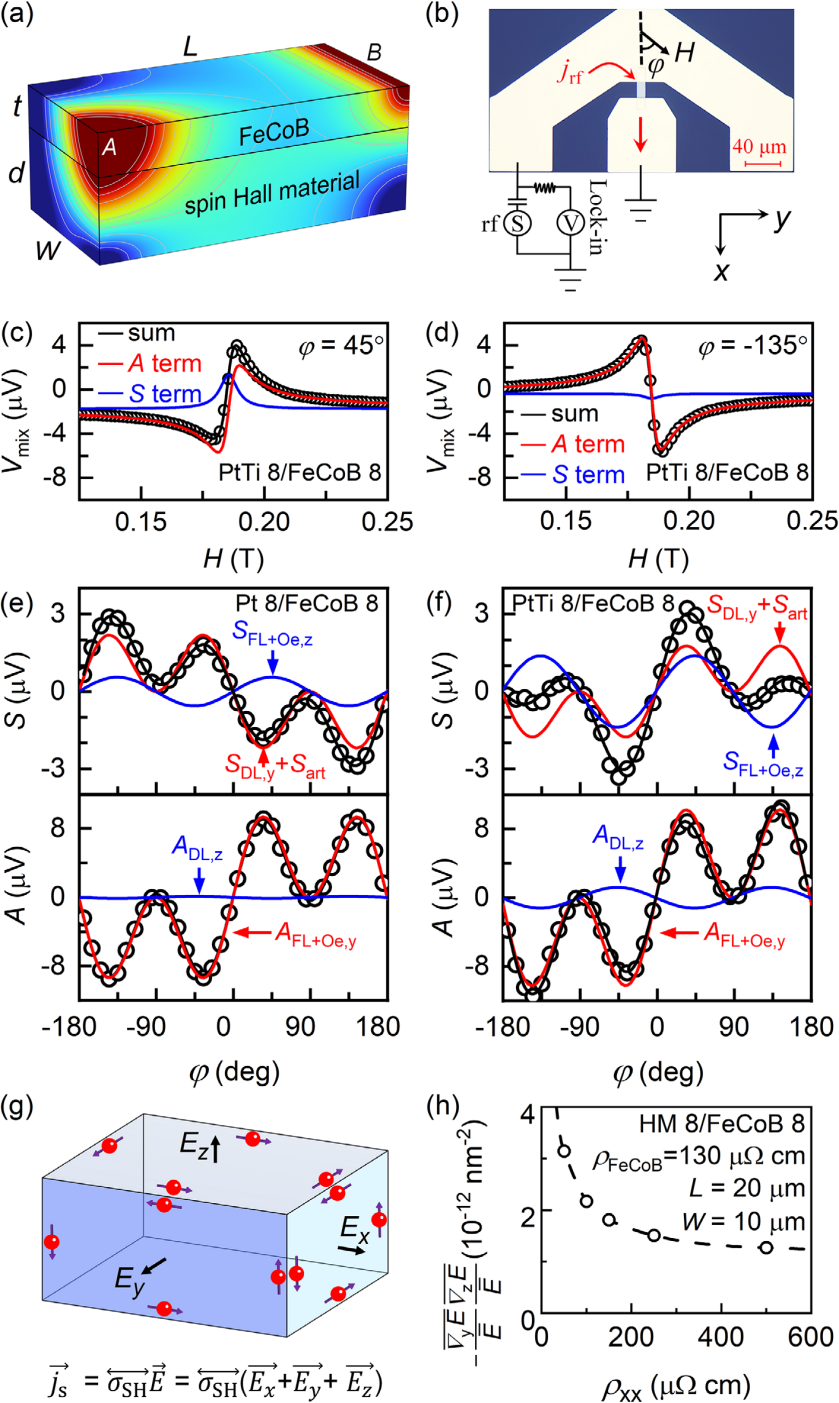
Enhancing z‐spin generation by alloying. a) Schematic depiction of the electric field magnitude distributions that indicate the transverse and perpendicular electric asymmetries in a spin Hall metal/FeCoB bilayer strip when a charge current is injected from corner A and flows out from side B. b) Optical microscopy image of a ST‐FMR device and the two‐terminal measurement configuration. Representative ST‐FMR spectra of the PtTi 8/FeCoB 8 device for the azimuth angle (*φ*) of the field at c) *φ* = 45° and d) *φ* = 135°. Dependences on *φ* of *S* and *A* for e) the Pt 8/FeCoB 8 and f) the PtTi 8/FeCoB 8 devices. g) Spin current generation by the spin Hall conductivity tensor $\overset{↔}{\sigma_{\mathrm{SH}}}$ and an arbitrary electric field $\vec{E}$. h) Finite‐element simulation result of ‐$\frac{\bar{\nabla_{y}E}}{\bar{E}}\frac{\bar{\nabla_{z}E}}{\bar{E}}$ versus the resistivity of the heavy metal (HM). The blue curves represent the contributions of *z* spins and perpendicular Oersted field, and the red curves represent the contributions of *y* spins and transverse Oersted field. In (c‐f) and (h), *L* = 10 µm, *W* = 20 µm.

Recently, the efficient generation of *z* spins has been demonstrated in Ta/Py bilayers that were sputter‐deposited on an oxidized silicon wafer by tuning the spin Hall conductivity tensor ($\overset{↔}{\sigma_{\mathrm{SH}}}$) of the Ta via electric asymmetries^[^ ^34^ ^]^ (see **Figure** **1**a for a simplified, schematic depiction of the electric field distribution that indicates the transverse and perpendicular electric asymmetries). However, Pt devices only generate minimal *z* spins^[^ ^12^ ^]^ likely because the spin Hall conductivity tensor of pure Pt is robust against electric asymmetries.^[^ ^34^ ^]^ Technologically, it is of particular importance to enhance *z* spin generation in Pt since it already has the giant spin Hall conductivity for *y* spins,^[^ ^46^ ^]^ low resistivity, high spin‐mixing conductance in contact with ferromagnets,^[^ ^47^, ^48^ ^]^ and sputter‐deposition on silicon oxide.^[^ ^46^ ^]^ On the other hand, enhancing the SOT exerted on the ferromagnet Fe_60_Co_20_B_20_ is particularly interesting for its direct relevance to magnetic tunnel junctions in memory and computing technologies.

Here, we, for the first time, report a giant enhancement of *z* spin generation in sputter‐deposited Pt_75_Ti_25_/Fe_60_Co_20_B_20_ (PtTi/FeCoB) heterostructures by alloying Pt with Ti and by engineering the electric asymmetry via the layer thicknesses and geometry of the device. We show that the *z* spins generated by the PtTi can enable highly efficient, deterministic, nearly full, record‐low‐power, external‐magnetic‐field‐free switching of the FeCoB with strong perpendicular magnetic anisotropy (PMA) and high coercivity (*H*_c_).

## 2 Results

### 2.1 Enhancing z and y Spin Generation by Alloying

Stimulated by the previous reports that the intrinsic spin Hall effect and thus the spin Hall ratio of Pt can be engineered significantly by alloying,^[^ ^46^, ^48^, ^49^, ^50^, ^51^, ^52^ ^]^ we attempt to enhance the *z* spin component of $\overset{↔}{\sigma_{\mathrm{SH}}}$ by alloying Pt with another metal Ti. Magnetic bilayers of Pt 8/FeCoB 8 and PtTi 8/FeCoB 8 are fabricated on 4‐inch silicon oxide substrates and patterned into spin‐torque ferromagnetic resonance (ST‐FMR) devices (see the optical microscopy image in Figure 1b). As shown in Figure 1c,d, the ST‐FMR spectrum (*V*_mix_) of each device is collected as a function of the in‐plane field (*H*) while fixing the azimuth angle (*φ*) of *H* relative to the rf current (the *x* direction) asymmetrically injected into the device using the two‐terminal geometry (Figure 1b). The ST‐FMR spectrum includes a symmetric (*S*) and anti‐symmetric (*A*) component, which can be separated from fits of the spectrum to the equation

(1) $$V_{mix}=S\frac{\Delta H^{2}}{\Delta H^{2}+{\left(H-H_{r}\right)}^{2}}+A\frac{\Delta H\left(H-H_{r}\right)}{\Delta H^{2}+{\left(H-H_{r}\right)}^{2}}$$

where ∆*H* is the resonance linewidth and *H*_r_ is the resonance field. In general, *S* and *A* vary with *φ* as:^[^ ^34^ ^]^

(2) $$\begin{array}{c} S=\left(S_{\mathrm{DL},y}+S_{\mathrm{art}}\right)\sin2\varphi \cos\varphi +S_{\mathrm{DL},x}\sin2\varphi \sin\varphi +S_{\mathrm{FL}+\mathrm{Oe},z}\sin2\varphi \end{array}$$

(3) $$A=A_{\mathrm{FL}+\mathrm{Oe},y}\sin2\varphi \cos\varphi +A_{\mathrm{FL},x}\sin2\varphi \sin\varphi +A_{\mathrm{DL},z}\sin2\varphi$$

In Equation (2), *S*_DL,_ *_y_* is the contribution from the dampinglike SOT of *y* spins from the spin Hall metal,^[^ ^22^, ^53^ ^]^ *S*_art_ includes possible bulk SOT of self‐induced *y* spins,^[^ ^54^, ^55^ ^]^ spin pumping‐inverse spin Hall voltage of the *y* spins and the Nernst voltage induced by Joule heating,^[^ ^56^ ^]^ *S*_DL,_ *_x_* is the dampinglike SOT of the *x* spins, and *S*_FL+Oe,_ *_z_* is from the field‐like torque of the *z* spins and the perpendicular Oersted field.^[^ ^10^ ^]^ In Equation (3), *A*_FL+Oe,_ *_y_* is from the field‐like torque of *y* spins and the transverse Oersted field, *A*_FL,_ *_x_* is the field‐like torque of the *x* spins, and *A*_DL,_ *_z_* is the damping‐like torque of the *z* spins.^[^ ^22^ ^]^ From the fits of the *φ* dependences of *S* and *A* to Equations (2) and (3) in Figure 1e, the Pt 8/FeCoB 8 exhibits a minimal dampinglike torque of *z* spins but a sizable *S*_FL+Oe,_ *_z_* signal predominantly from the perpendicular Oersted field, which is consistent with previous experiments on Pt/Co and Pt/Py devices.^[^ ^12^ ^]^ In contrast, the PtTi 8/FeCoB 8 device exhibits much greater *S*_FL+Oe,_ *_z_* and *A*_DL,_ *_z_* signals (Figure 1f), indicating the presence of significantly enhanced damping‐like and field‐like SOTs of *z* spins in the PtTi devices. There is no *x* spin signal (*S*_DL,_ *_x_* ≈ 0) in the studied devices as is usually the case.^[^ ^12^, ^34^ ^]^

Following the method we discuss in the next section, $\xi_{\mathrm{DL},z}^{j}$ is estimated as 0.0016 for the Pt 8/FeCoB device and 0.0119 for the PtTi 8/FeCoB device (*W* = 10 µm, *L* = 20 µm). According to the drift‐diffusion spin transport,^[^ ^46^, ^57^ ^]^ the SOT exerted on a light ferromagnet by a *y* or *z* spin current from the spin Hall effect can be written as $\xi_{\mathrm{DL},y(z)}^{j}$ = *T*_int_ *σ*_SH,_ *_y_*_(_ *_z_*_)_ *ρ_xx_*, where *T*_int_ is the spin transparency of the interface*, σ*_SH,_ *_y_*_(_ *_z_*_)_ the spin Hall conductivity element of *y* spins (*z* spins), and *ρ_xx_* the resistivity of the spin Hall metal. Since *T*_int_ is expected to be similar (≈0.50) for both the Pt 8 and PtTi 8 devices,^[^ ^48^, ^58^ ^]^ we attribute the enhancement of *z* spin generation by alloying Pt with Ti to a significant tuning of the spin Hall conductivity tensor of the Pt (i.e., the enhancement of *σ*_SH,_ *_z_*) and the enhancement of the resistivity (*ρ_xx_* is ≈ 100 µΩ cm for the 8 nm PtTi and 19 µΩ cm for the 8 nm Pt). We stress that $\overset{↔}{\sigma_{\mathrm{SH}}}$ must be altered by alloying because the 5 times resistivity enhancement cannot, by itself, explain the more than tenfold enhancement of *z* spin torque.

We also point out that the enhancement of the *z* spin generation cannot be attributed to any current tilting itself. Without altering the spin Hall conductivity tensor, a tilted current flow cannot generate any *z* spin current. As shown in Figure 1g, the spin current $\vec{J_{s}}$ generated by an arbitrary electric field $\vec{E}$ can be written as $\vec{J_{s}}$ = $\overset{↔}{\sigma_{\mathrm{SH}}}\;\vec{E}$ = $\overset{↔}{\sigma_{\mathrm{SH}}}\;(\vec{E_{x}}$+$\vec{E_{y}}+\vec{E_{z}}$), while none of the three components of the electric field ($\vec{E_{x}}$, $\vec{E_{y}},\vec{E_{z}}$) can generate a spin current both polarized and flowing in the *z* direction. Moreover, our finite‐element simulation in Figure 1h indicates that the electric field gradients (characterized as the product of relative transverse and perpendicular electric field gradients, $\frac{\bar{\nabla_{y}E}}{\bar{E}}\frac{\bar{\nabla_{z}E}}{\bar{E}}$, the simulation method is described in the Methods and ref. [34]), thus the tilting of the current flow, decreases rather than increases upon increase of the resistivity of the spin Hall metal.

### 2.2 Enhancing z Spin Generation by Electric Asymmetry Engineering

**Figure 2:**
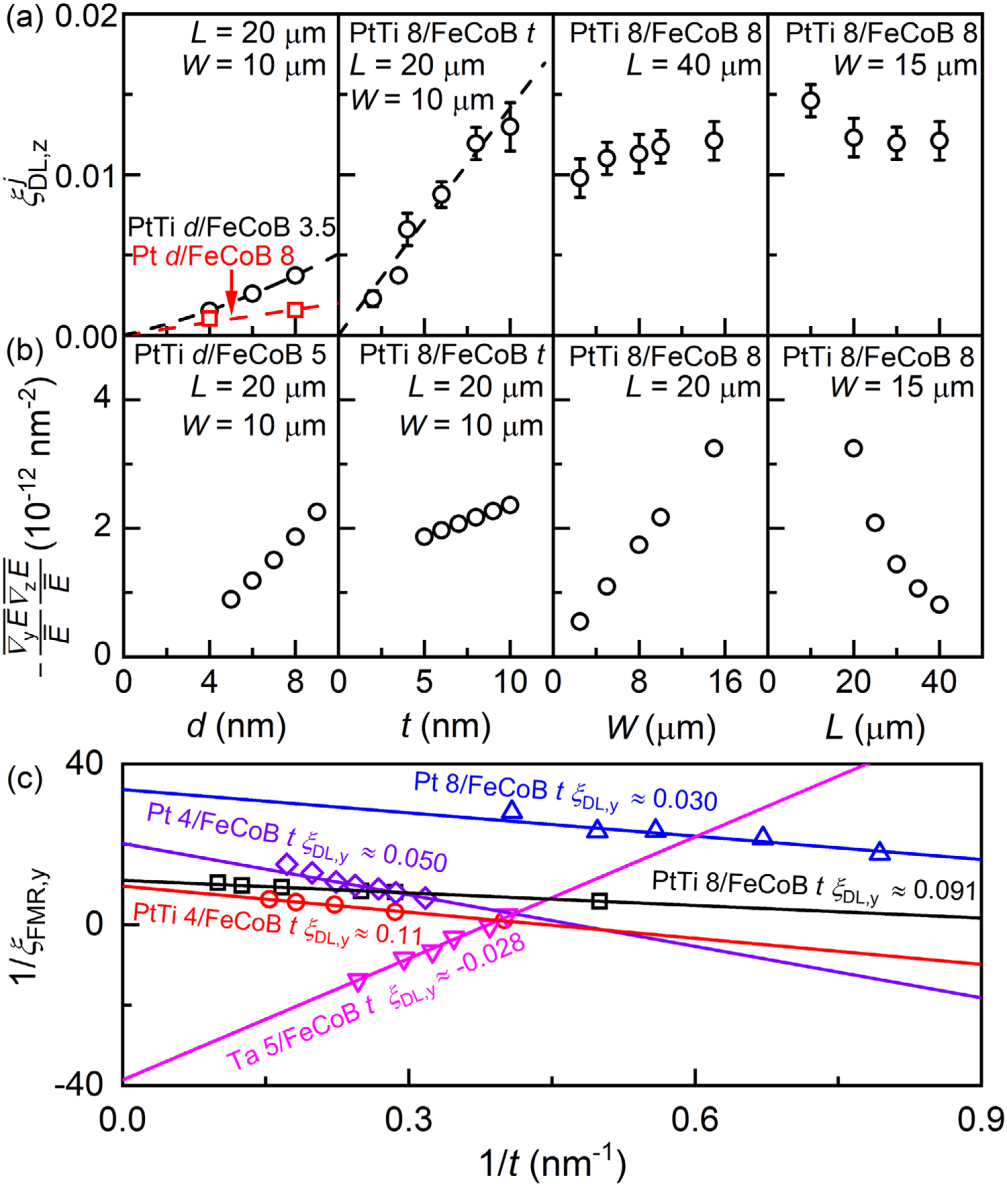
Spin‐orbit torque efficiencies of *y* spins and *z* spins. Dependences of a) $\xi_{\mathrm{DL},z}^{j}$ and b) ‐$\frac{\bar{\nabla_{y}E}}{\bar{E}}\frac{\bar{\nabla_{z}E}}{\bar{E}}$ of the PtTi *d*/FeCoB *t* devices on the PtTi thickness *d* (*t* = 3.5 nm, *W* = 10 µm, *L* = 20 µm), and the FeCoB thicknesses *t* for (*d* = 8 nm, *W* = 10 µm, *L* = 20 µm), the width *W* (*t* = *d* = 8 nm, *L* = 20 µm), and the width *L* (*t* = *d* = 8 nm, *W* = 15 µm). The red squares plot $\xi_{\mathrm{DL},z}^{j}$ for the Pt 8/FeCoB. c) 1/*ξ*_FMR,_ *_y_* versus 1/*t* for the PtTi 4/FeCoB *t*, PtTi 8/FeCoB *t*, Pt 4/FeCoB *t*, and Pt 8/FeCoB *t* (*W* = 10 µm, *L* = 20 µm).

We show below that the *z* spin generation can be significantly enhanced by engineering the electric symmetries of the device (Figure 1a) via the thickness of the spin Hall metal (*d*), the thickness of the magnetic layer (*t*), the width (*W*) and length (*L*) of the magnetic strip of the PtTi/FeCoB. The principle is simply that the emergence of the spin Hall conductivity of *z* spins is directly relevant to the electric symmetries of the devices (exactly zero in the absence of the vertical and/or longitudinal electric symmetries). **Figure** **2**a,b shows the experimental results of the damping‐like torque efficiency of the *z* spins ($\xi_{\mathrm{DL},z}^{j}$) and the finite‐element simulation results of the degree of the electric asymmetries as a function of *d*, *t*, *W*, and *L*. As discussed previously,^[^ ^34^ ^]^ $\xi_{\mathrm{DL},z}^{j}$ can be estimated following

(4) $$\;\xi_{\mathrm{DL},z}^{j}=\xi_{\mathrm{DL},y}^{j}\;A_{\mathrm{DL},z}/S_{\mathrm{DL},y}\sqrt{1+M_{\mathrm{eff}}/H_{r}}$$

where *M*_eff_ is the effective magnetization as determined from the rf frequency dependence of *H*_r_ (Figure S1, Supporting Information). The damping‐like SOT efficiency of *y* spins ($\xi_{\mathrm{DL},y}^{j}$) is 0.110 ± 0.001 for the PtTi 4/FeCoB *t* (*ρ_xx_* ≈ 120 µΩ cm for 4 nm PtTi), 0.091 ± 0.002 for the PtTi 8/FeCoB *t* (*ρ_xx_* ≈ 100 µΩ cm for 8 nm PtTi), 0.050 ± 0.001 for the Pt (4)/FeCoB and 0.030 ± 0.003 for the Pt 8/FeCoB *t* (*ρ_xx_* ≈ 19 µΩ cm for 8 nm Pt), as determined from the inverse intercept of the linear fit^[^ ^59^ ^]^ of 1/*ξ*_FMR,y_ versus 1/*t* (Figure 2c),

(5) $$\frac{1}{\xi_{\mathrm{FMR},y}}=\frac{1}{\xi_{\mathrm{DL},y}^{j}}\;\left(1+\frac{\hbar \xi_{\mathrm{FL},y}^{j}}{e\mu_{0}M_{s}td}\right)$$

here, *e* is the elementary charge, ℏ the reduced Planck's constant, *µ*_0_ the permeability of vacuum, *M*_s_ the saturation magnetization, $\xi_{\mathrm{FL},y}^{j}$ the fieldlike torque efficiency of *y* spins, and *ξ*_FMR,y_ is defined as^[^ ^59^ ^]^

(6) $$\xi_{\mathrm{FMR},y\;}\equiv \;\frac{S_{\mathrm{DL},y}}{A_{\mathrm{FL},y}\;}\frac{e\mu_{0}M_{s}td}{\hbar }\sqrt{1+M_{\mathrm{eff}}/H_{r}}$$

As shown in Figure 2a, $\xi_{\mathrm{DL},z}^{j}$ can be increased significantly by increasing the PtTi thickness, the FeCoB thickness, and the width, and by decreasing the length, which is qualitatively consistent with variations of the simulated geometry‐enhanced electric asymmetries in Figure 2b. We note that the simulated dependences of $-\frac{\bar{\nabla_{y}E}}{\bar{E}}\frac{\bar{\nabla_{z}E}}{\bar{E}}$ on *t*, *d*, *W*, and *L* in Figure 2d are quantitatively stronger or weaker than the experimental variations of $\xi_{\mathrm{DL},z}^{j}$ in Figure 2a. This could be attributed to the overlook of the contact resistance between the electrodes and the magnetic strip and the interface resistance at the HM/FM interfaces in the finite‐element analysis. It is also possible that the efficiency of the *z* spin generation is positively correlated to, but not exactly a linear function of $-\frac{\bar{\nabla_{y}E}}{\bar{E}}\frac{\bar{\nabla_{z}E}}{\bar{E}}$. We also note that the dependences of *S*_FL+Oe,_ *_z_*/*S*_DL,y_ on *d*, *t*, *W*, and *L* (Figure S2, Supporting Information) are quite similar to those of $\;\xi_{\mathrm{DL},z}^{j}$, suggesting an enhancement of field‐like torque of the *z* spins and perpendicular Oersted field. We attribute the observed enhancement of the z‐spin torques by the device geometry to an enhancement of the z‐spin Hall conductivity (*σ*_SH,_ *_z_*) by improved electric asymmetries. The strong dependences of the *z*‐spin torque on the layer thicknesses, lateral geometry, and contact of the device unambiguously reveal that the *z* spin torque is not from any interface effects, which is different from the mechanism of a previous report.^[^ ^33^ ^]^ The maximum value of $\;\xi_{\mathrm{DL},z}^{j}$ is 0.015 for the optimal PtTi 8/FeCoB 8 device with *W* = 15 µm and *L* = 10 µm. Further enhancement of $\;\xi_{\mathrm{DL},z}^{j}$ is possible if electric asymmetries could be enhanced by fine geometry optimization via, e.g., layer thicknesses and contact design.

### 2.3 Technological impacts

**Figure 3:**
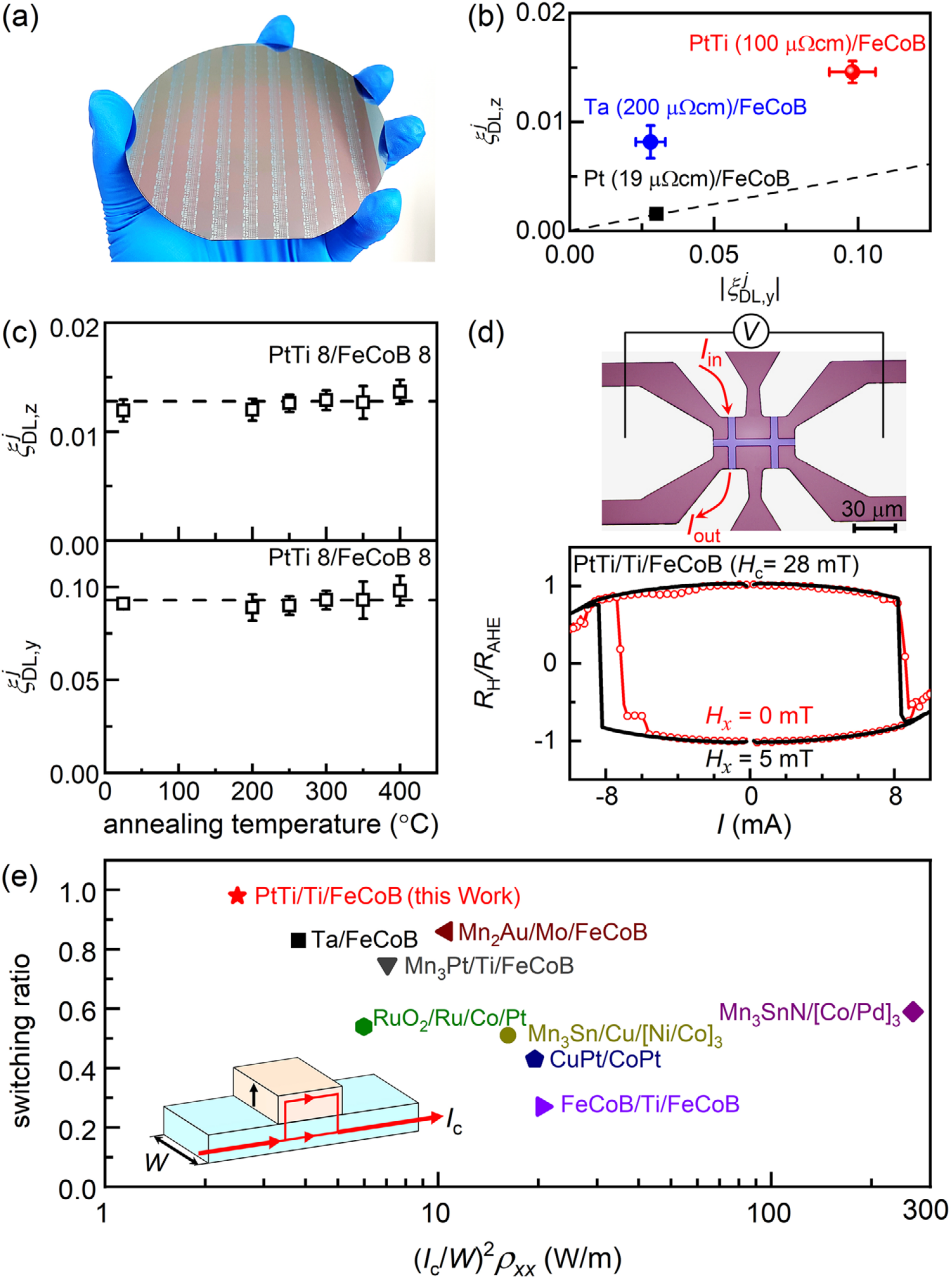
Technological importance. a) Wafer‐scale fabrication (4‐inch oxidized silicon wafer) of the z‐spin devices of PtTi/Ti/FeCoB heterostructure with strong perpendicular magnetic anisotropy and high coercivity. b) Comparison of $\;\xi_{\mathrm{DL},z}^{j}$, $\;\xi_{\mathrm{DL},y}^{j}$, and resistivity of the PtTi 8/FeCoB 8, Pt 8/FeCoB 8, and Ta 4/FeCoB 8.1 devices. c)$\;\xi_{\mathrm{DL},z}^{j}$ and $\;\xi_{\mathrm{DL},y}^{j}$ of the PtTi 8/FeCoB 8 as a function of the annealing temperature. In (b) and (c), *W* = 10 µm, *L* = 20 µm. d) Optical microscopy image of the Hall‐bar device, the C‐shaped current injection configuration, and current‐driven switching of the PtTi 5.6/Ti 0.8/FeCoB 1.3 with (black) and without external magnetic field (red), suggesting a complete field‐free switching. e) Comparison of the switching ratio and the power parameter of the representative *z*‐spin Hall‐bar devices, including the PtTi 5.6/Ti 0.8/FeCoB 1.3, Ta/FeCoB,^[^ ^34^ ^]^ in‐plane FeCoB/Ti/perpendicular FeCoB,^[^ ^24^ ^]^ CuPt/CoPt,^[^ ^10^ ^]^ Mn_3_Sn/Cu/[Ni/Co]_3_,^[^ ^31^ ^]^ Mn_3_Pt/Ti/FeCoB,^[^ ^12^ ^]^ RuO_2_/Ru/Co/Pt,^[^ ^30^ ^]^ Mn_2_Au/Mo/FeCoB,^[^ ^13^ ^]^ and Mn_3_SnN/[Co/Pd]_3_.^[^ ^27^ ^]^

Technologically attractive devices should simultaneously have good compatibility with large‐scale integration with CMOS and magnetic tunnel junctions, relatively low resistivity, high SOT efficiencies of *z* spins and *y* spins, deterministic energy‐efficient switching, and high coercivity at the same time. As shown in **Figure** **3**a, the PtTi‐based z‐spin device allows for wafer‐scale uniform sputter‐deposition on commercial Si/SiO_2_ substrates, which is critical for large‐scale integration into CMOS circuits and in sharp contrast to other field‐free strategies requiring antiferromagnetic or piezoelectric single crystals^[^ ^7^, ^10^, ^14^, ^15^, ^16^, ^26^, ^27^, ^28^, ^29^, ^30^, ^31^ ^]^ or the composition^[^ ^3^, ^17^, ^18^, ^19^ ^]^ or thickness wedges.^[^ ^3^, ^21^ ^]^ The PtTi‐based z‐spin devices with the magnetic FeCoB layer on the top also show great promise for integration with high‐performance magnetic tunnel junctions.^[^ ^1^, ^40^, ^60^, ^61^ ^]^

As summarized in Figure 3b, the PtTi 8/FeCoB 8 has much greater $\xi_{\mathrm{DL},z}^{j}$ and $\xi_{\mathrm{DL},y}^{j}$ than the Pt 8/FeCoB 8 and the Ta 5/FeCoB 8, but a factor of 2 smaller resistivity than the Ta device (200 µΩ cm). The combination of the high $\xi_{\mathrm{DL},z}^{j}$, $\xi_{\mathrm{DL},y}^{j}$, and low resistivity of the PtTi‐based z‐spin device should benefit the current and energy efficiencies, impedance, operation speed, and endurance of the SOT devices in memory and logic technologies. In Figure 3c, we further show that both $\;\xi_{\mathrm{DL},z}^{j}$ and $\;\xi_{\mathrm{DL},y}^{j}$ of the PtTi 8/FeCoB 8 do not degrade by annealing up to 400 °C, fulfilling the thermal stability requirement of CMOS integration. In Figure 3d, we demonstrate complete, deterministic switching of a typical PtTi 5.6/Ti 0.8/FeCoB 1.3 Hall‐bar device (with large PMA field of 490 mT, high coercivity *H*_c_ of 28 mT, and *M*_s_ of 1190 kA m^−3^, Figure S3, Supporting Information) in the absence of any external magnetic field by an in‐plane charge current of ≈ 7.9 mA (or current density of 1.9×10^7^ A cm^−2^ within the PtTi layer) injected in a “C‐shaped” contact configuration. Here, the 0.8 nm Ti spacer layer is used to enhance the PMA of the FeCoB layer. We note that the very small electric asymmetry required for *z* spin generation (Figure 2b) is not expected to induce any significant thermal concentration within the electrical contact or the spin Hall channel of practical devices (Figure 3d). We also find no degradation of the devices during the repeated switching measurements (Figure S4, Supporting Information).

However, the switching current density within the spin‐current generating layer (*j*_c_) is not always a fair indicator for the energy efficiency of practical nanodevices because, despite the width (*W*) of the device can be scaled down, the layer thicknesses may not be scaled for most structures due to the thickness dependences of *j*_c_, the SOTs, resistivity, current shunting into the ferromagnets. Thus, we parameterize the energy efficiency by (*I*_c_/*W*)^2^ *ρ_xx_* rather than simply *j*_c_^2^ *ρ_xx_*. As compared in Figure 3e, the PtTi /Ti/FeCoB device exhibits the lowest energy efficiency and the highest switching ratio among the stable perpendicular SOT devices when switched by *z* spin‐current in the absence of any external magnetic field, including Ta/FeCoB (*H*_c_ = 20 mT, electric asymmetries),^[^ ^34^ ^]^ in‐plane FeCoB/Ti/ perpendicular FeCoB (*H*_c_ = 2 mT, orthogonal trilayer),^[^ ^24^ ^]^ and CuPt/CoPt (*H*_c_ = 24.8 mT, low‐symmetry magnetic crystal),^[^ ^10^ ^]^ Mn_3_Sn/Cu/[Ni/Co]_3_ (*H*_c_ = 10.8 mT, low‐symmetry antiferromagnetic crystal),^[^ ^31^ ^]^ Mn_3_Pt/Ti/FeCoB (*H*_c_ = 6.1 mT, low‐symmetry antiferromagnetic single crystal),^[^ ^12^ ^]^ RuO_2_/Ru/Co/Pt (*H*_c_ = 40 mT, low‐symmetry altermagnetic crystal),^[^ ^30^ ^]^ Mn_2_Au/Mo/FeCoB (*H*_c_ = 82 mT, low‐symmetry antiferromagnetic crystal),^[^ ^13^ ^]^ and Mn_3_SnN/[Co/Pd]_3_ (*H*_c_ = 1.4 mT, low‐symmetry antiferromagnetic crystal).^[^ ^27^ ^]^

The PtTi/Ti/FeCoB device with a high coercivity (*H*_c_ = 28 mT) is also advantageous over the low‐coercivity architectures of FeCoB/Ti/FeCoB (2 mT),^[^ ^24^ ^]^ Mn_3_SnN/[Co/Pd]_3_ (1.4 mT),^[^ ^27^ ^]^ Mn_3_Sn/Cu/[Ni/Co]_3_ (10.8 mT),^[^ ^31^ ^]^ and Mn_3_Pt/Ti/FeCoB (6.1 mT).^[^ ^12^ ^]^ This is because a perpendicular SOT device must have a sufficiently high coercivity to achieve the required magnetic stability in the environment, although it linearly increases the switching current within the switching models of macrospin or domain wall depinning.^[^ ^1^ ^]^

## 3 Conclusion

We have demonstrated giant enhancement of *z* spin generation by alloying the strongest spin Hall metal Pt with Ti and by electric symmetry engineering, without the need for any thickness or composition gradients. The optimal Pt_75_Ti_25_/FeCoB devices exhibit a factor of 8 and 3 enhancement in the damping‐like SOT efficiencies of *z* spins and *y* spins, and enable complete deterministic switching of perpendicularly magnetized FeCoB devices at record‐low power consumption. The combination of the highly efficient simultaneous generation of *z* and *y* spin currents without the need for any thickness or composition gradients, relatively low resistivity, wafer‐scale uniform sputter‐deposition on silicon oxide, and excellent thermal stability of exceeding 400 °C make the Pt_75_Ti_25_/FeCoB heterostructure a very compelling candidate for scalable, energy‐efficient, field‐free, and CMOS‐compatible spin‐torque technologies. These results establish an effective strategy to develop high‐performance z‐spin generators, especially by alloying and electric asymmetry engineering. We have also clarified that, without altering the spin Hall conductivity tensor, a tilted current flow can never generate any *z* spin current because none of the three components of the electric field ($\vec{E_{x}}$, $\vec{E_{y}},\vec{E_{z}}$) can generate a spin current both polarized and flows in the *z* direction.

## 4 Experimental Section

### Sample Fabrications

Samples for this work include spin‐torque ferromagnetic resonance (ST‐FMR) devices of PtTi 4, 6, 8/FeCoB 2–10, Pt 8/FeCoB 8, and Ta 5/FeCoB 8 with in‐plane magnetic anisotropy and a PtTi 5.6/Ti 0.8/FeCoB 1.3 Hall‐bar device with perpendicular magnetic anisotropy (PtTi = Pt_75_Ti_25_, FeCoB = Fe_60_Co_20_B_20_, the numbers are layer thicknesses in nanometers). Each device was sputter‐deposited on an oxidized silicon substrate and protected by a MgO 1.6/TaO*_x_* 1.6 bilayer. The Pt_75_Ti_25_ layers are grown by co‐sputtering using Pt and Ti targets. The base pressure during deposition is below 8 × 10^−9^ Torr. All the devices are patterned using photolithography and ion milling, followed by the deposition of Ti 5/Pt 150 as the electrical contacts. Annealing experiments were performed in a vacuum at different temperatures for 30 min.

### Finite‐Element Analysis

The electric asymmetries and the relative electric field distributions are simulated by finite‐element analysis for a heavy metal/FeCoB bilayer device (with the length *L*, the width *W*, the FeCoB thickness *t*, and the HM resistivity *ρ_xx_*) covered by two contact pads with a dimension of 20µm × 40µm × 150 nm and a resistivity of 24 µΩ cm. The distribution of charge current density/electric field is calculated following the current continuity equation $\nabla \cdot j_{c}+\frac{\partial \rho }{\partial t}=0$ and the boundary condition of ***n*** ∙ ***j***_c_ = 0, where ***j***_c_ = ***E*** */ρ_xx_* is the charge current density, ρ is the charge density, *n* is the normal direction of the device boundary. The resistivity of the FeCoB is fixed at 130 µΩ cm following our resistivity calibration. The element size is 200 nm (length)×200 nm (width)×0.16 nm (thickness).

### ST‐FMR Analysis

The magnitudes of *S*_DL,_ *_y_*, *S*_DL,_ *_z_*, *A*_FL,y_, and *A*_DL,z_ in Equations (2) and (3) correlate to the magnitudes of *H*_DL,y_, *H*_DL,z_, *H*_FL,y_, and *H*_FL,z_ as

(7) $$S_{\mathrm{DL},y}=I_{\mathrm{rf}}C_{\mathrm{MR}}H_{\mathrm{DL},y}$$

(8) $$S_{\mathrm{DL},z}=I_{\mathrm{rf}}C_{\mathrm{MR}}\left(\hbar /2e\right)H_{\mathrm{DL},z}$$

(9) $$\begin{array}{ccc} A_{\mathrm{FL},y} & = & I_{\mathrm{rf}}C_{\mathrm{MR}}\left(H_{\mathrm{FL},y}+H_{\mathrm{Oe},\;y}\right)\sqrt{1+M_{\mathrm{eff}}/H_{r}}\\ & = & I_{\mathrm{rf}}C_{\mathrm{MR}}\left(\frac{\hbar }{2e}j_{\mathrm{HM}}\frac{\xi_{\mathrm{FL},y}}{\mu_{0}M_{s}t_{\mathrm{FM}}}+\frac{j_{\mathrm{HM}}d_{\mathrm{HM}}}{2}\right)\sqrt{1+M_{\mathrm{eff}}/H_{r}} \end{array}$$

(10) $$A_{\mathrm{DL},z}=I_{\mathrm{rf}}C_{\mathrm{MR}}H_{D}\sqrt{1+M_{\mathrm{eff}}/H_{r}}$$

where *C*_MR_ is a coefficient related to the magnetoresistance of the magnetic layer, and *H*_Oe,_ *_y_* is the Oersted field.

## Conflict of Interest

The authors declare no conflict of interest.

## Supporting information



Supporting Information

## Data Availability

The data that support the findings of this study are available from the corresponding author upon reasonable request.
