# Supplementary material for: Enhancing z Spin Generation in Trivial Spin Hall Materials for Scalable, Energy‐Efficient, Field‐Free, Complete Spin‐Orbit Torque Switching Applications
Source: Adv Sci (Weinh). 2025 Jul 25;12(39):e07581. doi: 10.1002/advs.202507581 (PMC12533401; doi:10.1002/advs.202507581)
Supplement: Supplementary file 1 — Supporting Information [file ADVS-12-e07581-s001.pdf]

ADVANCED SCIENCE
Open Access

Supporting Information

for *Adv. Sci.*, DOI 10.1002/advs.202507581

Enhancing *z* Spin Generation in Trivial Spin Hall Materials for Scalable, Energy-Efficient, Field-Free, Complete Spin-Orbit Torque Switching Applications

*Qianbiao Liu and Lijun Zhu**

Supporting information for

Enhancing z spin generation in trivial spin Hall materials for scalable, energy-efficient, field-free, complete spin-orbit torque switching applications

Qianbiao Liu[1], Lijun Zhu[1,2]*

[1]*State Key Laboratory of Semiconductor Physics and Chip Technologies, Institute of Semiconductors, Chinese Academy of Sciences, Beijing 100083, China*

[2]*Center of Materials Science and Optoelectronics Engineering, University of Chinese Academy of Sciences, Beijing 100049, China*

**ljzhu@semi.ac.cn*

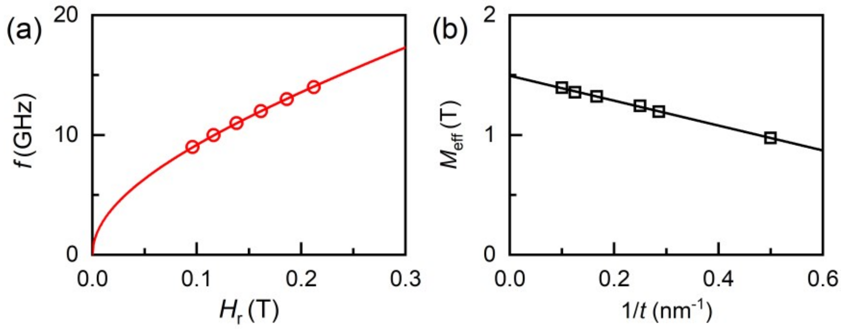

Fig. S1 Determination of the effective magnetization. (a) Rf frequency (f) vs the resonance field (H_r) of the PtTi 8/FeCoB t (t =2nm). The solid line represents the best fit of the data to the Kittel's equation, i.e., $f = (2\pi/\gamma)\sqrt{H_r(H_r + M_{\mathrm{eff}})}$. (b) Linear dependence of the effective magnetization field (M_{eff}) on the inverse thickness of the FeCoB layer in the PtTi 8/FeCoB t samples, the intercept of which indicates the saturation magnetic of 1190 kA/m^3.

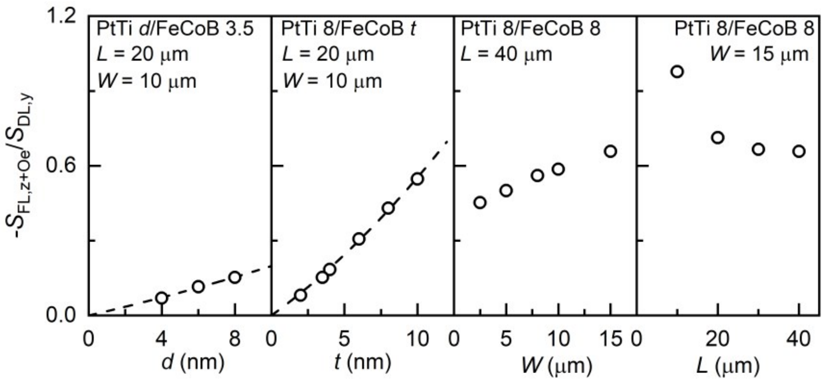

Fig. S2 Fieldlike torque of z spins and perpendicular Oersted field. Dependences of -$S_{\mathrm{FL+Oe},z}/S_{\mathrm{DL,y}}$ of the PtTi d/FeCoB t devices on the PtTi thickness d (t = 3.5 nm, W=10 μm, L=20 μm), and the FeCoB thicknesses t for (d = 8 nm, W=10 μm, L=20 μm), the width W ($t = d$ = 8 nm, L=20 μm), and the width L ($t = d$ = 8 nm, W=15 μm). Here, $S_{\mathrm{DL,y}}$, which is proportional to the rf current and the efficiency of the dampinglike SOT of the y spins ($\xi^{j}_{\mathrm{DL,y}}$), normalizes $S_{\mathrm{FL+Oe},z}$ to be at the same current density. The dashed curves in (a) guide the eyes.

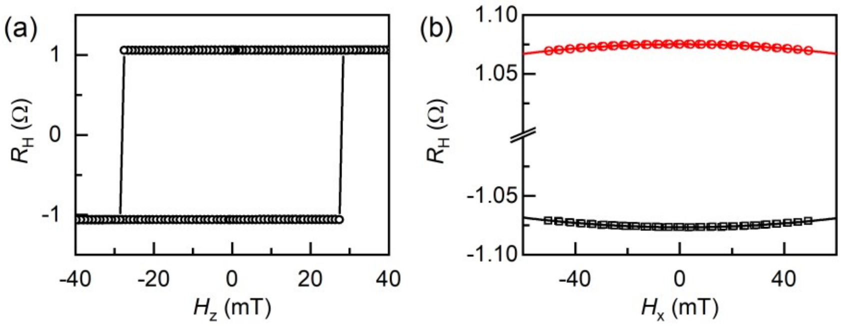

Fig. S3 Coercivity and perpendicular magnetic anisotropy for the PtTi 5.6/Ti 0.8/FeCoB 1.3 device. Dependences of the anomalous Hall resistance (R_H) on (a) the perpendicular magnetic field (H_z) and (b) in-plane magnetic field (H_x), suggesting a coercivity (H_c) of 28 mT, an effective perpendicular anisotropy field (H_k) of 490 mT, and anomalous Hall resistance of 1.075 Ω. Solid curves in (b) represent fits of the data to a parabolic function.

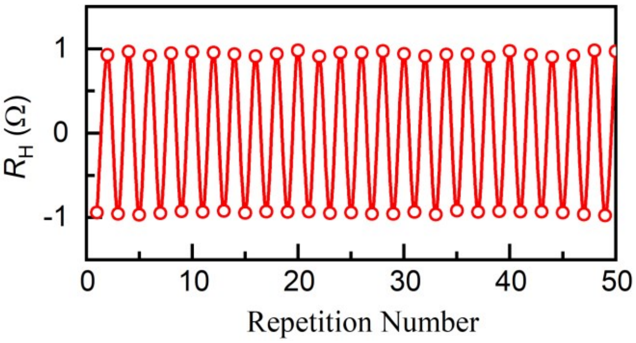

Fig. S4 Anomalous Hall resistane of the PtTi 5.6/Ti 0.8/FeCoB 1.3 device plotted as a function of the number of the switching current pulses, indicating no degradation of the device upon repeated switching.
